# Genome Sequencing of Chromosome 1 Substitution Lines Derived from Chinese Wild Mice Revealed a Unique Resource for Genetic Studies of Complex Traits

**DOI:** 10.1534/g3.116.033902

**Published:** 2016-09-06

**Authors:** Fuyi Xu, Tianzhu Chao, Yingming Liang, Kai Li, Shixian Hu, Maochun Wang, Yuxun Zhou, Hongyan Xu, Junhua Xiao

**Affiliations:** * College of Chemistry, Chemical Engineering, and Biotechnology, Donghua University, Shanghai 201620, China; † The Laboratory of Genetic Regulators in the Immune System, Xinxiang Medical University, Henan 453003, China; ‡ Department of Biostatistics and Epidemiology, Medical College of Georgia, Augusta University, Georgia 30912

**Keywords:** Chinese wild mice, chromosome substitution, whole genome sequencing, genetic polymorphisms, haplotype

## Abstract

Mouse resources such as Collaborative Cross, outbred stocks, Hybrid Mouse Diversity Panel, and chromosome substitution strains have been instrumental to many progresses in the studies of complex traits genetics. We have established a population of chromosome 1 (Chr 1) substitution lines (C1SLs) in which donor chromosomes were derived from Chinese wild mice. Genome sequencing of 18 lines of this population showed that Chr 1 had been replaced by the donor chromosome. About 4.5 million unique single nucleotide polymorphisms and indels were discovered on Chr 1, of which 1.3 million were novel. Compared with sequenced classical inbred strains, Chr 1 of each C1SL had fivefold more variants, and more loss of function and potentially regulatory variants. Further haplotype analysis showed that the donor chromosome accumulated more historical recombination events, with the largest haplotype block being only 100 kb, and about 57% of the blocks were <1 kb. Subspecies origin analysis showed that these chromosomes had a mosaic genome structure that dominantly originated from *Mus musculus musculus* and *M. m. castaneus* subspecies, except for the C57BL/6J-Chr1^KM^ line from *M. m. domesticus*. In addition, phenotyping four of these lines on blood biochemistry suggested that there were substantial phenotypic variations among our lines, especially line C57BL/6J-Chr1^HZ^ and donor strain C57BL/6J. Further gene ontology enrichment revealed that the differentially expressed genes among liver-expressed genes between C57BL/6J and C57BL/6J-Chr1^HZ^ were enriched in lipid metabolism biological processes. All these characteristics enable C1SLs to be a unique resource for identifying and fine mapping quantitative trait loci on mouse Chr 1, and carrying out systems genetics studies of complex traits.

Mouse as a model organism plays an important role in the dissection of the genetic basis of complex traits involving both genetic and environmental factors. Traditional F2 population for linkage studies with two extreme opposite phenotype strains could have sufficient power but generally lack resolution (Flint *et al.* 2005; Flint and Eskin 2012). Therefore, new methods and resources have been developed to improve the efficiency of quantitative trait loci (QTL) mapping and gene identification.

Over the last several decades, genome-wide association studies (GWAS) in humans have discovered thousands of genetic loci related to complex diseases or traits (Tim *et al.* 2014), and a similar strategy can be implemented in mouse populations. The Hybrid Mouse Diversity Panel (HMDP), which consists of ∼100 inbred strains, including 29 classical inbred (CI) strains and 71 recombinant inbred strains, has been used in mouse GWAS and systems genetics (Ghazalpour *et al.* 2012; Bennett *et al.* 2015). It has been estimated that this panel can detect a single nucleotide polymorphism (SNP) that contributes to 5% of the overall phenotypic variation in a 2.6–2.7 Mb genomic region (Bennett *et al.* 2010). Recent studies have shown that expanding mice strains and genotyping density would further improve its power and resolution (Rau *et al.* 2015). The Collaborative Cross (CC) panel aims to generate >1000 recombinant inbred strains derived from eight diverse mouse strains, which could provide a mapping resolution of ∼2 Mb (Buchner and Nadeau 2015). It is noteworthy that CC parental strains include three wild-derived strains, thereby dramatically increasing the genetic and phenotypic diversity of this resource. However, only 73 strains have been made publicly available so far (http://csbio.unc.edu/CCstatus/). Despite this limitation, investigators have successfully used pre-CC and completed CC strains in genetic studies of complex traits (Aylor *et al.* 2011; Buchner and Nadeau 2015). Another valuable resource is outbred stocks (OS). Different from HMDP and CC, this resource is intended to maximize genetic diversity through carefully planned breeding. The accumulation of recombination events over time in OS markedly decreases linkage disequilibrium (LD) compared to inbred strains (Yalcin and Flint 2012). It should be noted that individual mice within an OS are not genetically identical. Therefore, every individual needs to be genotyped when performing association studies. Some currently available OS include heterogeneous stock (Valdar *et al.* 2006; Ahlqvist *et al.* 2011) and the diversity outbred stock (Svenson *et al.* 2012; French *et al.* 2014).

Unlike HMDP, CC, and OS, in which genotype–phenotype associations are conducted on a heterogeneous genetic background, chromosome substitution strains (CSSs), constructed by transferring a single full-length chromosome from the donor strain onto the genetic background of the recipient strain by repeated backcrossing, are pursued to construct an homogeneous genetic background, except for the donor chromosome (Nadeau *et al.* 2000). Therefore, QTL on the donor chromosome can be relatively easier to detect. However, because a CSS panel is derived from just two mouse strains, they have less genetic diversity relative to CC, HMDP, and OS. Another problem is that if a QTL has been identified in a particular chromosome, further fine mapping is generally needed to narrow down the QTL, through the construction of congenic strains (DeSantis *et al.* 2013; Kato *et al.* 2014).

Wild mice are another promising recourse for fine mapping and gene identification. Previous studies have shown that they harbor more genetic variations, accumulate more historical recombination events, and LD decays considerably more rapidly with physical distance than laboratory populations. Therefore, strong associations can be detected within regions <100 kb, which can help to achieve single gene resolution (Laurie *et al.* 2007). Combining the respective advantages of CSSs and the wild mice resource depicted above, we proposed a new strategy to fine mapping and gene identification using a population of specific CSSs (Xiao *et al.* 2010). Several years ago, we began constructing a population of chromosome 1 substitution lines (C1SLs). Briefly, 30 mice were used as chromosome 1 (Chr 1) donors, including 24 wild mice captured across China, one C57BL/6J-Chr1^KM^ (KM) line mouse and five inbred strains, with C57BL/6J (B6) as the recipient strain. The high genetic diversity on the replaced chromosome and the homogeneous background enable promising applications for fine mapping and gene identification. In this study, we carried out the whole genome sequence analysis for 18 lines of this population. As expected, there were large amounts of genetic variations and smaller haplotype blocks distributed on Chr 1, and a homogeneous genome background on other chromosomes. In addition, phenotyping some of these lines also revealed that they had high phenotypic diversity. All these results indicated that this population could serve as a unique and complementary resource for identifying and fine mapping QTL on mouse Chr 1, and carrying out systems genetics studies of complex traits.

## Materials and Methods

### Mice

In the 18 sequenced C1SLs, 17 donors were wild mice captured from different localities in China, the other donor KM mouse and recipient strain B6 were purchased from Shanghai SLAC Laboratory Animal Co., Ltd. (Shanghai, China). Briefly, the donor male was mated with a B6 female to produce hybrid F1, followed by seven generations of backcrossing using 29 SNPs evenly spaced on Chr 1 to assist selection, then brother/sister mating to create C1SLs. All mice were housed in a climate-controlled facility (temperature: 18–22°, humidity: 40–60%) with a 12 hr light/12 hr dark cycle, and allowed *ad libitum* access to water and a chow diet (M01-F25; Shanghai SLAC Laboratory Animal Co., Ltd.).

### High-throughput sequencing

Genomic DNA was extracted from the tail tissue of each mouse using an AxyPrep Multisource Genomic DNA Miniprep Kit (Axygen) as the manufacturer’s default protocol described. Purified genomic DNA was sheared and size selected (300–500 bp). Paired end sequencing (2 × 150 bp) was carried out with an Illumina X Ten instrument (Illumina Inc., San Diego, CA) by WuXi AppTec (Shanghai, China) according to the manufacturer’s protocol, except that the KM line was sequenced with Illumina Hiseq2500 (PE125) on two lanes.

### Quality control and read alignment

Raw reads were quality filtered with NGS QC Toolkit v2.3 (Patel and Mukesh 2012) to remove reads containing >30% low-quality (*Q* < 20) bases. Filtered reads were aligned to the mouse reference sequence (GRCm38/mm10) using speedseq v0.03a (Chiang *et al.* 2015) align module, which implemented BWA-MEM for read alignment, SAMBLASTER for in-memory duplicate marking, and Sambamba for multithreaded sorting and indexing to produce BAM alignment files for downstream analyses.

### Variants identification and annotation

Variants identification were conducted individually for each line. SAMtools v1.2 mpileup and BCFtools v1.2 call function (Li *et al.* 2009) were used for SNP and indel calling, and the parameters “-t DP,DV,DP4,SP,DPR,INFO/DPR -E -Q 0 -pm3 -F0.25 –ugf ” and “-mv -f GQ,GP -p 0.99” were used for mpileup and call function. In order to identify high-quality variants data, variants were filtered using the vcf-annotate function in vcftools v0.1.12b (Danecek *et al.* 2011). The following parameters were used for all lines: −*H* −*f* +/*d* = 5/*q* = 20/*w* = 2/*a* = 5. For maximum depth, BLD, DX, FX, HZ, JD, QP, SMX, SY, TW, and TZ lines were set to 100; CM, KM, YP, ZC, and ZZ1 were set to 120; and the remaining lines, including YX, PD, and ZZ2, were set to 140.

We used the sv module, which implemented lumpy-sv in speedseq software, to identify structural variants (SVs). Raw SVs were then filtered using the following criteria: calling quality >100, and length between 50 and 100,000 bp for deletion and <3,000,000 bp for duplication and inversion.

Ensembl Variant Effect Predictor (VEP) v80 (McLaren *et al.* 2010) was used to annotate the variants, and the SIFT algorithm was used to predict whether a missense variants would have a deleterious effect in a protein-coding gene.

### Sensitivity and specificity of variant calls

We randomly selected 81 genomic segments spread over Chr 1 on ZZ2. Primers were designed by NCBI primer designing tool, Primer-BLAST. Each segment was PCR amplified and sequenced by capillary sequencing technology. After trimming the low-quality bases from two sides of each sequence manually, the trimmed sequences were mapped to the mouse reference genome to call SNP. This SNP data set was compared with SNP calls from the same regions using next generation sequencing. If an SNP was present in the Sanger-sequenced SNP calls but not present in our next generation sequencing SNP calls, this SNP was considered to be false negative. The reverse was considered to be false positive.

### Subspecies origin analysis

SNP and indel information of 36 sequenced inbred strains were downloaded from the mouse genome project (MGP) at Sanger institute (http://www.sanger.ac.uk/science/data/mouse-genomes-project). Chr 1 consensus sequences for three wild-derived strains [CAST/EiJ (CAST), PWK/PhJ (PWK), and WSB/EiJ (WSB)] and 18 C1SLs were constructed using SAMtools consensus parameter. The Chr 1 sequence was divided into 19,548 10-kb blocks. The percent similarity of each block of the Chr 1 sequence of each C1SL to the sequences of WSB, CAST, and PWK was then calculated. The subspecies with the highest percentage similarity was inferred as the subspecies origin for each block.

### Haplotype analysis

We used the haplotype block definition based on the four gamete rule (Wang *et al.* 2002) to infer haplotype blocks. A total of 127 randomly selected Chr 1 regions were used in the analysis. For each region, haplotype analysis was performed with haploview v4.2 (Barrett *et al.* 2005) with the four gamete block (GAM) output option.

### Blood biochemistry analysis

Blood biochemistry was measured in four C1SLs (CM: *n* = 8, HZ: *n* = 6, KM: *n* = 11, and ZZ2: *n* = 4) and recipient strain B6 (*n* = 15). Male mice were first maintained on a chow diet (M01-F25; Shanghai SLAC Laboratory Animal Co., Ltd.) for 8 wk, then fed with 10% fat diet (D12450B; Research Diets, New Brunswick, NJ) until killed at 20 wk of age. All mice were fasted for 4 hr in the morning before blood collection.

Blood from the retro-orbital sinus was collected in 1.5-ml tubes containing EDTA, and centrifuged at 2500 × *g* for 15 min. Blood serum was frozen at −20° until assay. Low-density lipoprotein cholesterol (LDL-C), high-density lipoprotein cholesterol (HDL-C), triglycerides, cholesterol (CHOL), total protein (TP), albumin, total bilirubin, alanine aminotransferase (ALT), aspartate aminotransferase (AST), alkaline phosphatase, glucose (GLU), and creatinine concentrations were measured using a biochemical blood analyzer (Hitachi 7180; Hitachi, Tokyo, Japan) by Sino-British SIPPR/B&K Lab Animal Ltd. (Shanghai, China). Mice were killed by cervical dislocation.

### RNA isolation and expression profiling

Four HZ and four B6 male mice, examined for blood biochemistry were also used to perform transcription profiling. Liver tissues were collected and total RNAs were immediately extracted with RNAiso Plus reagent [Takara Biotechnology (Dalian) Co., Ltd, Liaoning, China], according to the manufacturer’s protocol, followed by quantification with NanoDrop 2000c. They had an A260/A280 of 1.8–2.0. For each line, RNA from two mice were pooled and assayed on one Affymetrix Mouse Transcriptome Assay 1.0 chip. CEL files were imported into Expression Console v1.4 (Affymetrix). The SST-RMA algorithm was used to background correct, normalize, and summarize all expression values. Then, the output CHP files were imported into Transcriptome Analysis Console v3.0 (Affymetrix) to conduct gene-level differential expression analysis.

### Evaluation of genome background contamination

For each line, the genome was divided into 100-kb nonoverlapping intervals. We used SNP density to estimate contaminations on other chromosomes. We calculated the mean and SD of the number of SNPs per 100-kb region on Chr 1, and established a lower boundary of mean −2 SD. If the number of SNPs per 100-kb region on other chromosomes was larger than this boundary, this interval was considered to be a wild-type residual contamination.

### Ethics statement

All mice were maintained under specific pathogen-free conditions, according to the People’s Republic of China Laboratory Animal Regulations, and the study was conducted in accordance with the recommendations of, and was approved by the Laboratory Animal Committee of Donghua University.

### Data availability

All raw reads were submitted to NCBI Sequence Read Archive under the accession number SRP066591. Expression data can be obtained from the Gene Expression Omnibus database (accession no. GSE81078). This mouse resource is available to the community of mouse geneticists and can be obtained upon request.

## Results

### Data generation and variants discovery

In this study, C1SLs from 17 Chinese wild mice and one KM mouse were whole genome–sequenced with Illumina sequencing-by-synthesis technology, with a target insert size of 300–400 bp, and 1965 Gb of data were obtained. Sequence reads were filtered for quality and then aligned to the mouse reference genome (GRCm38/mm10) using the Burrows–Wheeler Aligner implemented in speedseq software, which achieved at least 30× coverage for all lines (Table 1 and Supplemental Material, Table S1).

**Table 1:** An overview of the mapping statistics and Chr 1 variants called from C1SLs

Line	Raw Data (Gb)	Coverage	SNPs	Private SNPs	Indels	Private Indels	Deletion	Duplication	Inversion
C57BL/6J-Chr1^BLD^ (BLD)	105.78	33.38	1,649,522	52,394	252,898	10,658	4672	79	27
C57BL/6J-Chr1^CM^ (CM)	120.7	40.01	1,723,794	43,128	276,719	11,658	5089	91	38
C57BL/6J-Chr1^DX^ (DX)	105.85	34.16	1,727,183	138,380	258,376	23,732	4814	83	34
C57BL/6J-Chr1^FX^ (FX)	93.1	30.12	1,655,800	29,596	248,619	6855	4650	69	40
C57BL/6J-Chr1^HZ^ (HZ)	106.3	33.58	1,592,121	43,347	243,930	8991	4511	72	41
C57BL/6J-Chr1^JD^ (JD)	98.43	31.04	1,637,950	31,717	249,763	7317	4621	63	32
C57BL/6J-Chr1^KM^ (KM)	126.34	36.29	495,093	135,477	93,122	25,265	1568	40	16
C57BL/6J-Chr1^PD^ (PD)	135.3	47.36	1,617,372	52,102	259,382	12,123	4771	98	36
C57BL/6J-Chr1^QP^ (QP)	91.6	29.39	1,680,439	45,986	252,079	8768	4687	73	43
C57BL/6J-Chr1^SMX^ (SMX)	92.65	30.2	1,701,840	35,669	260,102	8435	4802	80	39
C57BL/6J-Chr1^SY^ (SY)	96.8	30.41	1,699,121	66,972	254,768	13,605	4762	74	35
C57BL/6J-Chr1^TW^ (TW)	95.1	32.01	1,698,145	25,736	256,637	6095	4809	83	38
C57BL/6J-Chr1^TZ^ (TZ)	101.88	32.84	1,621,359	37,581	249,672	8107	4649	79	31
C57BL/6J-Chr1^YP^ (YP)	107.74	35.19	1,730,297	51,049	265,384	10,337	4916	87	44
C57BL/6J-Chr1^YX^ (YX)	127.9	44.54	1,715,406	23,054	275,745	7151	5050	112	44
C57BL/6J-Chr1^ZC^ (ZC)	111.8	38.9	1,645,630	29,850	265,876	8276	4805	94	40
C57BL/6J-Chr1^ZZ1^ (ZZ1)	118.38	37.49	1,711,022	17,537	261,709	4698	4926	90	38
C57BL/6J-Chr1^ZZ2^ (ZZ2)	128.9	45.01	1,713,734	31,041	274,396	8838	5044	94	39
Total	1964.55	641.92	29,015,828	890,616	4,499,177	190,909	83,146	1461	655

Private variants are line-specific variants.

**Figure 1:**
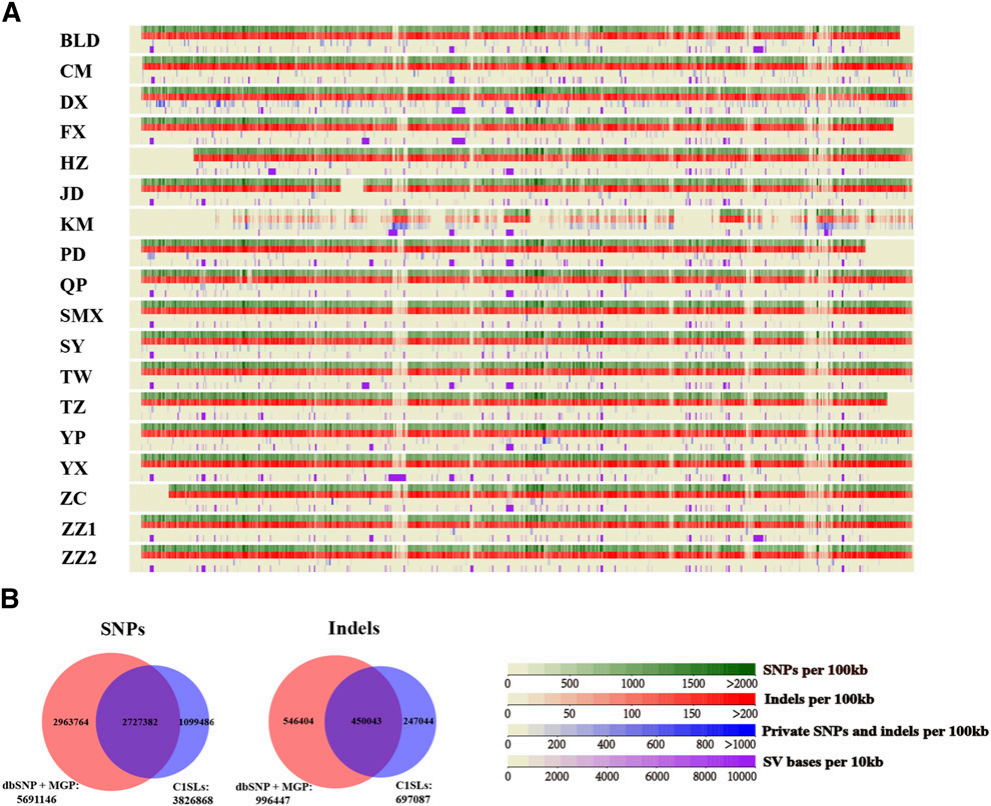
Overview of variants called from C1SLs. (A) Distribution of SNPs, indels, private variants, and structural variants on Chr 1 in C1SLs. (B) Venn diagram of comparison between C1SLs and MGP and dbSNP142 data sets of identified SNPs and indels on Chr 1.

We used SAMtools/BCFtools to call SNPs and indels from the reads of each sample, and these variants were custom filtered (see *Materials and Methods*). Overall, ∼29 million SNPs and 4.5 million indels (1–50 bp) were identified from the 18 Chr 1 sequences (Table 1 and Figure 1A). For SNP genotypes, we found that 97.4% were homozygous and 2.6% were heterozygous on average (Table S2), the latter most likely arising from true heterozygote loci due to incomplete fixation of the inbred strain, false-positive calls due to regions of copy number variation, sequencing errors, and mapping errors in highly repetitive genomic regions, as previously described (Keane *et al.* 2011). The sensitivity and specificity of our variant calls were established using 23-kb bases of DNA from the ZZ2 line. We sequenced 81 PCR segments spread over Chr 1 using Sanger sequencing. A total of 352 SNPs were detected. None of the SNPs were missed by whole genome sequencing, resulting in a false negative rate of 0% in our next-generation SNP calls. Two SNPs identified by whole genome sequencing were not found in Sanger sequencing, which gave a false positive rate of 0.57%.

Compared with CI strains, we found large amounts of genetic variants in each line. There were, on average, 1.68 million SNPs and 0.25 million indels in our sequenced lines, except for strain KM (0.5 million SNPs and 0.09 million indels). However, for sequenced 36 strains from MGP, only 0.31 million SNPs were called for each strain, except for seven wild-derived inbred strains (Table S3) (Keane *et al.* 2011). SNP density across Chr 1 was 8.2 SNP/kb (SD = 1.2), which was fivefold more than CI strains (1.6 SNP/kb, SD = 0.5) (Table S3). When the 18 lines were compared, each line carried relatively few private variants (mainly <3% of all variants called in each line), except for KM (27%) and DX (8%) (Table 1), indicating that these two had large divergence from the other lines. Next, we merged all the variants from these lines and identified ∼4.5 million (4,523,955) unique variants, including 3,826,868 SNPs and 697,087 indels. Further comparison with entries in MGP and NCBI dbSNP142 variant data sets identified 2.7 million SNPs (71%) as known, and the remaining SNPs (1,099,486; 29%) were classified as novel. For indels, 450,043 (64.5%) had been described previously (Figure 1B).

To identify SVs from the information of split and discordant reads and changes in mapped read depth, lumpy-sv implemented in speedseq software was used. We called 0.09 million SVs, including 83,146 deletions (>50 bp), 1461 duplications, and 655 inversions (Table 1 and Figure 1A).

### Variants annotation

To understand the functional consequences of the variants called in these 18 donor chromosomes, we annotated these variants using the VEP from Ensembl (Table 2). Within the total set of 4.5 million variants, 0.47% (21,335) were in protein-coding regions, among which 38.3% (8181) were annotated to be amino acid (AA)-altering from 1002 protein-coding genes. Of the 8181 AA-altering variants, 130 indels caused a disruption of the translational reading frame (Table S4); 64 were predicted as premature truncation of the protein due to gain of stop codons, whereas 24 were predicted as loss of stop codons (Table S5). Apart from the AA-altering variants, the other potentially functional categories included: variants with the potential to disrupt splicing events (3,841; 0.08%); variants in 3′ and 5′ untranslated regions (UTRs) with the potential to regulate protein translation (49,163; 1.1%); those within 5 kb upstream or downstream of transcription start or end sites with possible roles on transcriptional regulation (1,038,410; 23%). As shown in Table 2, we also found that a large amount of novel variants fell into coding sequences and potentially regulatory regions, including 3009 missense variants, 45 stop gains, six stop loss, and 65 frameshift variants potentially having loss of function effects, and those including 1036 splicing variants, 14,154 3′ and 5′ UTR variants, and 303,537 5-kb upstream or downstream variants with potentially regulatory effect.

**Table 2:** Predicted functional consequence of SNPs and indels

Consequence	SNPs	Novel SNPs	Indels	Novel Indels
Coding Sequence Variant				
stop_gained	64	44	2	1
stop_loss	24	6	2	0
missense_variant	7959	3009	0	0
synonymous_variant	12,914	3323	0	0
frameshift_variant	NA	NA	130	65
inframe_insertion	NA	NA	85	26
inframe_deletion	NA	NA	135	56
coding_sequence_variant	7	0	13	8
Noncoding Sequence Variant				
start_loss	21	6	3	0
splice_donor_variant	115	37	29	11
splice_acceptor_variant	89	23	30	15
splice_region_variant	3072	821	506	125
stop_retained_variant	17	8	0	0
mature_miRNA_variant	20	11	19	11
5_prime_UTR_variant	5496	1763	875	310
3_prime_UTR_variant	35,358	9771	7434	2310
non_coding_transcript_exon_variant	84,594	22,389	13,702	4249
intron_variant	1,526,474	426,038	297,901	102,640
NMD_transcript_variant	278,249	74,313	55,018	17,982
non_coding_transcript_variant	785,325	214,776	151,618	51,692
upstream_gene_variant	406,576	115,964	83,207	28,457
downstream_gene_variant	456,092	127,304	92,535	31,812
intergenic_variant	1,889,179	555,948	323,620	117,902

Consequences were predicted using VEP and gene models from Ensembl version 80. Detail explanations of the consequences can be found on the VEP main webpage (http://useast.ensembl.org/info/docs/tools/vep/). It should be noted that one SNP or indel may have one or more consequences. Novel SNPs or indels are defined as variants that were not in MGP and dbSNP142 data sets.

**Figure 2:**
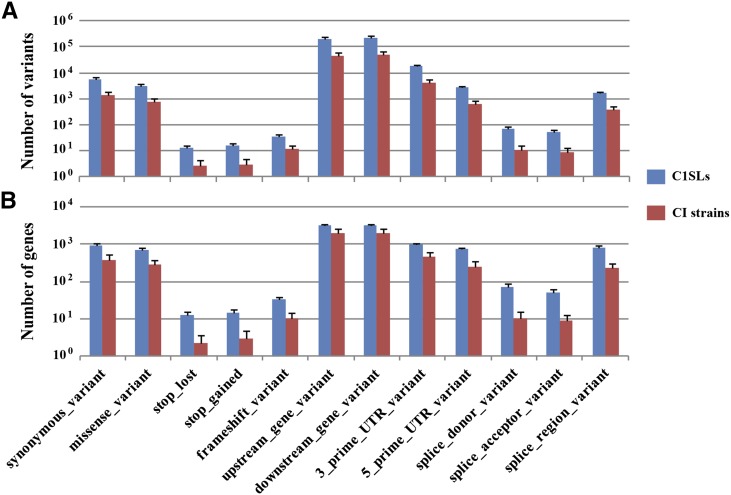
Comparison of potentially functional variants on chromosome between C1SLs and 31 MGP-sequenced CI strains. (A) Number of short variants in each categories. (B) Number of genes involved in each categories. All comparisons were statistically significant using a two-sample *t*-test (*P* < 0.01).

Another important question was whether there were more functional variants on Chr 1 for each line than the previously sequenced CI strains. To address this question, we compared variant distribution and number of involved genes with published data in those potentially functional categories. The results from this analysis showed that C1SLs had 4.9-fold (SD = 1.0) more variants in each category compared with MGP-sequenced CI strains. The number of genes involved were 3.8-fold (SD = 1.8) more (Figure 2). For the number of variants per gene in each category, we found that there were more variants in potentially regulatory regions, especially in upstream or downstream gene regions and 3′ UTR regions.

Among the protein-coding genes with AA-altering SNPs, 483 (Table S6) genes were predicted to have deleterious missense SNPs (SIFT score < 0.05). We annotated these genes and those with stop gain or loss and frameshift genes together to the KEGG pathway using the Database for Annotation, Visualization, and Integrated Discovery (DAVID) web-based tools (Huang da *et al.* 2009). This analysis resulted in 138 genes from 60 known pathways (Table S7). The first interesting finding was that 13 genes were involved in immune-related pathways, including the complement and coagulation cascades pathway and natural killer cell–mediated cytotoxicity pathway. We also found that there were 19 genes participating in olfactory transduction, most of which were olfactory receptor genes. Other pathways involving relatively more genes were cytokine–cytokine receptor interaction (seven genes) and adhesion molecules (seven genes).

There were 268 large deletions involving 157 genes. However, among them, only 30 were protein-coding genes, and others were pseudogenes or predicted genes. We also found 24 stop loss deletions, affecting 11 protein-coding genes.

### Haplotype block analysis

To capture the haplotype structure of the donor chromosomes, we randomly selected 126 nonoverlapping intervals in which each interval contained about 10,000 SNPs, on average 0.5 Mb per region, with sizes ranging from 0.3 to 1 Mb, and completely encompassing a 64 Mb region of the Chr 1 sequences. For each region, the haplotype block analysis was performed according to the principle of the four gamete rule (Wang *et al.* 2002).

**Figure 3:**
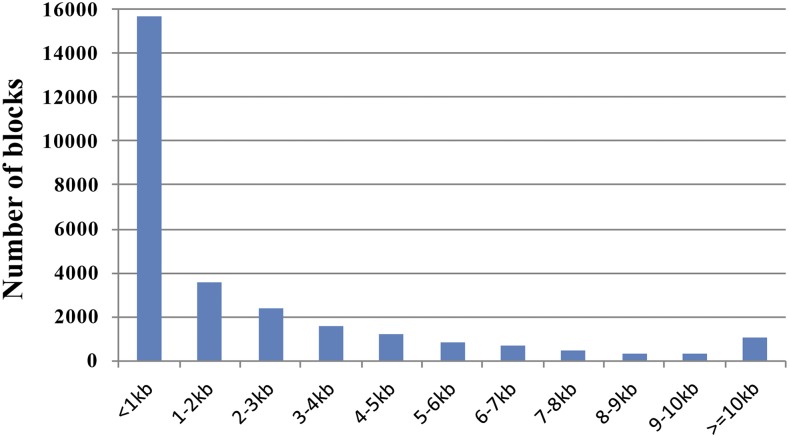
Distribution of haplotype block length across Chr 1 of C1SLs.

A total of 28,105 blocks were identified on these regions. The number of blocks varied for each region, ranging from 127 to 982. The average number of blocks was 221. There were six regions with an extremely large number of blocks (>88; mean + 2 SD of block number per 100 kb), indicating that these regions had many recombination events and may be recombination hotspots. From the block length distribution, nearly 56% of blocks were <1 kb in length, only 4% (1081 blocks) were ≥10 kb, and the largest block was 103.8 kb, while other blocks were between 1 and 10 kb (Figure 3). These results suggest that the haplotype blocks of the donor chromosomes are consistent with the previous study, where the haplotype blocks were mainly <100 kb (Laurie *et al.* 2007).

### Subspecies origin

We examined the ancestral subspecies origin of the 18 donor chromosomes, expecting to identify a mosaic of segments that could be assigned to one of three distinct lineages: *Mus musculus domesticus*, *M. m. musculus*, and *M. m. castaneus*. To assign subspecies origins to genomic intervals of the 18 lines, three wild-derived inbred strains, WSB, PWK and CAST, respectively, were chosen to represent the three lineages. We segmented each line’s Chr 1 sequence into 19,548 10-kb blocks and compared sequence similarity for each block with the corresponding sequence from WSB, PWK, and CAST to infer the subspecies origin for each segment. In this study, for each C1SL, we considered the regions with <5 SNP differences between this line and any two wild-derived inbred strains to be of undetermined origin, and excluded regions were considered to be subspecies that introgressed among those three wild-derived strains (Yang *et al.* 2007) (Table S8). This left an average of 63.2% (SD = 1.8%) of Chr 1 regions (Table S9) in each line for similarity analysis.

**Figure 4:**
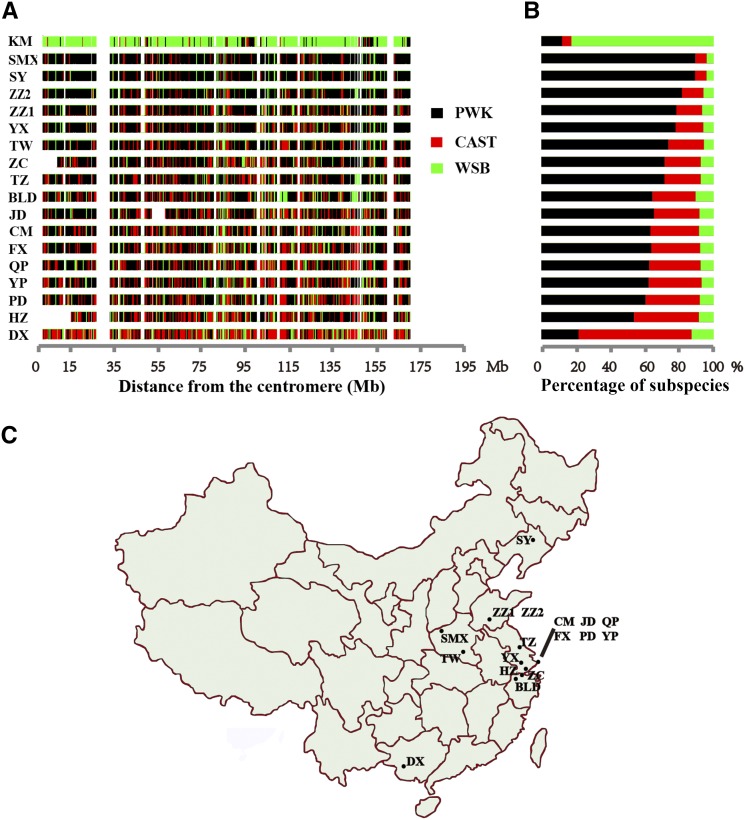
Subspecies origin of Chr 1 of C1SLs. (A) Subspecies assignments for the 18 donor chromosomes. Each 10-kb interval is shown as a vertical bar of a color that reflects its subspecies origin. Intervals without color are regions of intersubspecies introgression or of undetermined status. (B) Contribution of the three main subspecies lineages in Chr 1 of C1SLs. (C) Wild mice collection sites in China.

The results showed that all lines were a mosaic of regions that had all three subspecies origins (Figure 4A). However, each subspecies’ contribution was quite different (Figure 4B). KM had a large number of blocks with high sequence similarity to WSB, consistent with a previous report that KM mice are derived from Swiss mice (Zhang *et al.* 2007), which originated from *M. m. domesticus*. For the other 17 lines captured from different locations in China (Figure 4C), the sequences were mainly originated from *M. m. musculus* (68%, SD = 15%) and *M. m. castaneus* (25%, SD = 13%), which explained 93% (SD = 2%) of the Chr 1 sequence. A previous study found that there were two mouse subspecies, *M. m. musculus* and *M. m. castaneus*, in China, with the former mainly located in the north and the latter in the south (Xiao *et al.* 2010). However, we did not observe this distinct geographic distribution. Nonetheless, we found a consistent trend in the subspecies distribution. For example, the sequence of SY, which was from north of China, was largely contributed by *M. m. musculus* (89%) whereas the sequence of DX, which was from south of China, was largely contributed by *M. m. castaneus* (66%).

### Phenotypic diversity

**Figure 5:**
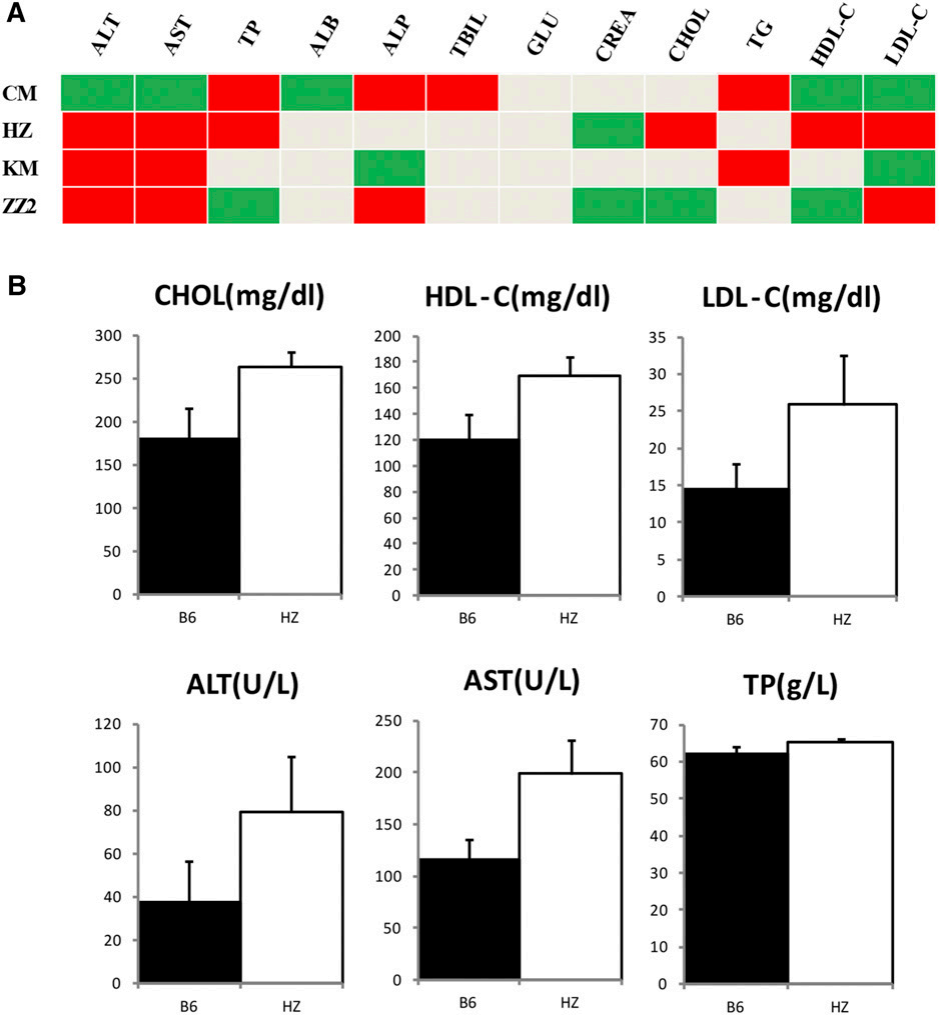
Comparison of phenotypes between C1SLs and recipient strain B6. (A) Comparison of 12 biochemistry indexes between CM, HZ, KM, and ZZ2, and recipient strain B6. Red: *P* < 0.01; green: *P* < 0.05. (B) Comparisons of six significant indexes between HZ and B6.

An ideal mouse resource for QTL mapping should not only have extensive genetic diversity, but also high phenotypic diversity. For CSSs, they should have some phenotypic differences from the recipient strain. In order to determine the phenotypic diversity, we examined blood chemistry indexes in four lines (CM, HZ, KM, and ZZ2) and recipient strain B6. Of the 12 indexes assayed, all except GLU had some significant differences between B6 and these four lines (Figure 5A). HZ exhibited differences in six indexes (TP, ALT, AST, CHOL, HDL-C, and LDL-C), the most among these four lines (Figure 5B). Interestingly, three blood lipid indexes (CHOL, HDL-C, and LDL-C) were significantly higher in HZ than B6, suggesting that HZ may have lipid metabolism disorders. We assayed gene expression levels in liver tissues to determine if these differences were reflected on intermediate phenotypes (gene expression levels). This assay contained 65,956 mouse genes, including 36,703 noncoding RNAs. After quality control and normalization, we identified 434 differentially expressed genes (fold change > 2 and *P* < 0.05), in which 130 genes were upregulated and 304 were downregulated compared to B6. Among these genes, 94 (22%) were located on Chr 1 whereas others were distributed on other chromosomes (Figure S1). Next, we performed gene ontology enrichment analysis using DAVID online tools (Huang da *et al.* 2009). This analysis yielded a list of 183 genes (excluding noncoding and unmapped genes). The results showed a significant enrichment of 32 PANTHER biological process categories. Table 3 lists the top 10 enrichment categories, most of which were associated with lipid metabolism, such as CHOL biosynthetic and metabolic process, sterol biosynthetic and metabolic process, and lipid biosynthetic process.

**Table 3:** Top 10 enriched gene ontology terms from a list of 183 genes differentially expressed between B6 and HZ

Term	Count	Percentage of Total	Fold Enrichment	*P*-Value
GO:0055114:oxidation reduction	27	16.4	5.2	4.07279E−12
GO:0016126:sterol biosynthetic process	6	3.6	25.9	2.91818E−06
GO:0006695:cholesterol biosynthetic process	5	3	28.1	2.56385E−05
GO:0016125:sterol metabolic process	7	4.2	11.8	2.65501E−05
GO:0008203:cholesterol metabolic process	6	3.6	11.1	0.000194489
GO:0006694:steroid biosynthetic process	6	3.6	10.9	0.000207962
GO:0006720:isoprenoid metabolic process	5	3	13.2	0.000526616
GO:0008299:isoprenoid biosynthetic process	4	2.4	23.5	0.000603412
GO:0008202:steroid metabolic process	7	4.2	5.6	0.001470541
GO:0008610:lipid biosynthetic process	9	5.5	4.1	0.00153588

All genes in the mouse genome were used as the background to determine enrichment. *P*-values were obtained using a modified Fisher’s exact test.

### Quality control of Chr 1 and genetic background

**Figure 6:**
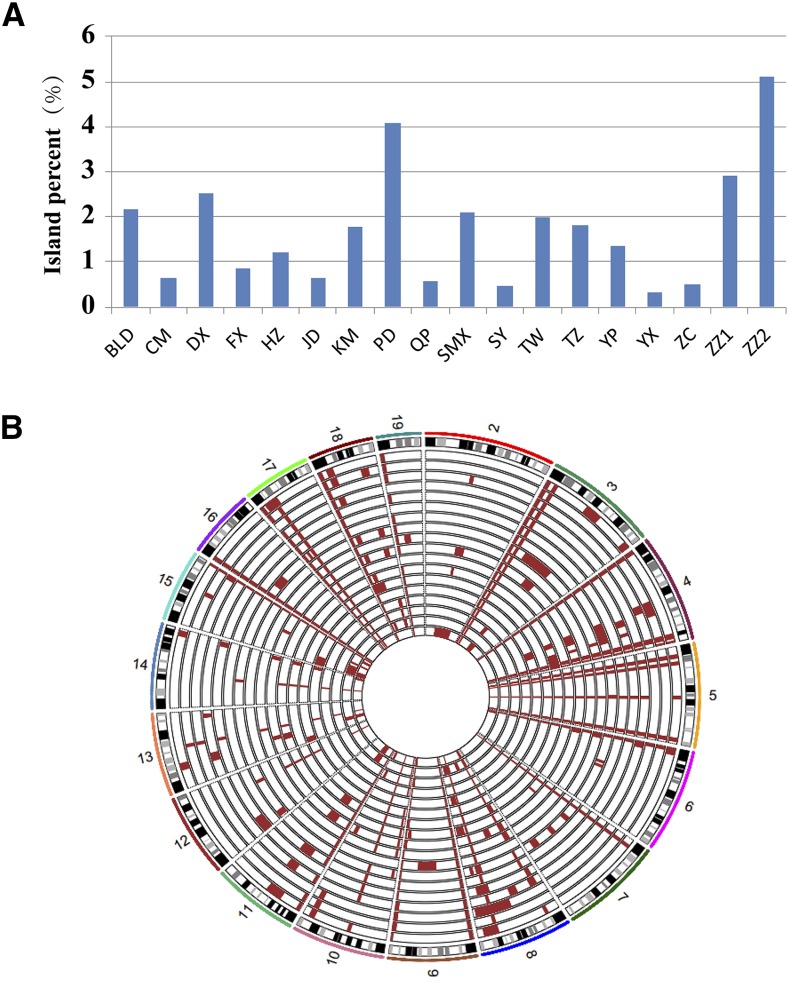
Wild island contamination across genome background for C1SLs. (A) Overall wild island contamination for each line. (B) Wild island distribution across the genome background (chromosomes 2 - 19). Brown bars represent wild islands. The C1SLs from inside out are ZZ2, ZZ1, ZC, YX, YP, TZ, TW, SY, SMX, QP, PD, KM, JD, HZ, FX, DX, CM, and BLD.

An ideal CSS should carry an intact donor chromosome and >99% of other genetic background identical to the recipient strain. Theoretically, with more generations of backcrosses, the genome background becomes increasingly pure while the probability of recombination event on the donor chromosome also increases (Gregorova *et al.* 2008). Among C1SLs, two lines, ZC and HZ, had a recombination on Chr 1 proximal sequence. As a result, these two lines’ Chr 1 proximal sequences became the B6 sequence. Similarly, the Chr 1 distal sequences of four lines (PD, TZ, FX, and BLD) were replaced by the B6 sequence (details in Table S10). For JD, we found a 5.7 Mb B6 sequence in the middle of Chr 1 (Table S10). For the other chromosomes from the genetic background, the residual contamination varied among these lines, ranging from 0.5 to 5.1% with an average of 1.7%, and six lines had >2% contamination (Figure 6A). As shown in Figure 6B, the wild island distributed across all autosomes, but some chromosomes such as chromosomes 4, 8, and 18 harbored more islands. Some islands were very large in certain lines. For example, BLD had a 7-Mb island on chromosome 3, DX had a 54-Mb island on chromosome 8, and ZZ2 had a 66-Mb island on chromosome 2. In addition, we also found that there were some overlapping islands in all of the 18 lines.

## Discussion

In 2008, we began a project to construct a population of C1SLs. After years of hard work, this project is near completion. In the present study, we performed whole genome sequencing on C1SLs from 17 Chinese wild mice and one KM mouse. We detected the genetic polymorphisms and examined their genetic structure, including their subspecies origin and haplotype structure. We also selected some lines to test the blood biochemical indexes. With all these genome sequences and limited phenotype data, we assessed the value of these lines for the genetic studies of complex traits, and for QTL fine mapping and detecting new QTL on Chr 1.

For the genetic studies of complex traits, high genetic diversity is essential for identifying causative genes. However, CI strains are originated from a limited progenitor, which constraints their genetic diversity (Yang *et al.* 2011). Recent whole genome sequencing of 36 inbred strains showed that most of the identified variants were from wild-derived strains (Keane *et al.* 2011). The SNP density for CI strains was only 1.6 SNP/kb (SD = 0.5) which was far less than wild-derived strains. Consistent with previous studies that wild mice harbor more genetic variants (Laurie *et al.* 2007; Salcedo *et al.* 2007), in our sequenced lines, we completely identified 4.5 million SNPs and indels on donor chromosomes, of which 1.3 million were not in the MGP and NCBI mouse dbSNP142 data sets. We identified nearly 1.6 million SNPs for each line, which was fivefold more than those sequenced CI strains (0.31 million). Further annotation also identified many loss of functional variants, especially among the novel variants (Table 2).

Another essential factor for the dissection of complex traits is mapping resolution. Recent progress in mouse resources have achieved a resolution of a ∼2 Mb interval containing roughly 10–20 genes (Buchner and Nadeau 2015), although the actual resolution varies with other factors, such as gene density. Other complementary methods, such as gene expression analysis and coexpression networks, can sometimes reduce the number of candidates to just one or a few genes and inform the underlying pathways (Mesner *et al.* 2014). Through haplotype analysis across these 18 lines, we found that the sizes of most haplotype blocks in our lines were <100 kb, with nearly 57% blocks <1 kb. This indicates that single gene resolution may be achieved when conducting association studies with C1SLs.

Unlike laboratory mice maintained under well controlled environments, wild mice have spread around, and adapted to, many different environmental pressures. Therefore, they should exhibit considerably more phenotypic variations (Phifer-Rixey and Nachman 2015). In this study, we found most of our assayed blood chemistry indexes had significant differences with B6. The HZ line showed significantly higher blood lipids (HDL-C, LDL-C, and CHOL) and liver damage–related indexes (TP, ALT, and AST). This was also reflected on expression levels of blood lipid metabolism–related genes. Through functional annotations of the identified genetic variants, we found 13 genes with deleterious coding sequence variants associated with immune-related pathways, including the complement and coagulation cascades pathway and natural killer cell–mediated cytotoxicity pathway, indicating that they have some differences in susceptibility of infectious diseases with B6. Indeed, there are reports that wild mice have different infectious disease susceptibility (Turner and Paterson 2013). In addition, these lines also harbored more functional variants on olfactory transduction genes, which may be related to their adaption to the environment. All these findings suggest that these newly built C1SLs can be used for the genetic analysis of many different phenotypes.

Our C1SLs also provide a potentially new resource for gene regulation and gene–gene interaction studies. Due to their special genetic makeup, for each gene or regulatory locus, there are at least two haplotypes and may be up to 19 from these mice lines, which may lead to improved power in gene regulation studies. In our sequenced lines, we found large amount of variants located in regulatory regions, suggesting *cis* regulation may exist for these nearby genes. Another finding was that nearly two-thirds of differentially expressed genes in the liver (between B6 and HZ) were located outside Chr 1. This difference may be triggered by the genetic variants on Chr 1 through *trans* regulations. Thus, this unique resource may provide more regulation information on the genes located on Chr 1.

The house mouse, *M. musculus*, consists of three principal subspecies, with native populations of *M. m. musculus* in Eastern Europe and Asia, *M. m. castaneus* in Southeast Asia and India, and *M. m. domesticus* in Western Europe and the Middle East (Boursot *et al.* 1993). CI strains are predominantly derived from *M. m. domesticus*, which accounted for about 94.3% of genome sequences (Yang *et al.* 2011). Our subspecies origin analysis on Chr 1 confirmed that nearly 93% sequences were from *M. m. musculus* and *M. m. castaneus*. The overall contribution for two subspecies for each line are consistent with the geographical distribution of subspecies in China. Thus, these newly constructed C1SLs have three subspecies of origin: for Chr 1, they predominantly originated from *M. m. musculus* and *M. m. castaneus*, while other chromosomes were mainly of *M. m. domesticus* origin.

Theoretically, ≥10 generations of backcrossing are required for purifying the CSS genome background (Nadeau *et al.* 2012), which is very time-consuming and laborious. In order to shorten the inbreeding time, we backcrossed seven generations in our lines. Through whole genome sequencing, we found that some lines were nearly purified with B6 background (CM, FX, JD, QP, SY, YX, and ZC), whereas others had <5% residual contamination in the genome background with wild islands. The residual contamination is mainly caused by the limited number of backcrosses. It may also be due to positive selection for these wild islands, which means that once these wild islands are replaced by B6 sequences, it may cause genetic incompatibility, leading to problems such as reproductive isolation (Ma *et al.* 2016). However, these limited islands can be easily traced through SNP genotyping to determine if the fixation status is B6 or wild allele in the future intercrossing, which can be used to guide further research.

Until now, nearly 1000 QTL have been mapped onto mouse Chr 1. However, only a small number of the underlying causative genes have been identified (Eppig *et al.* 2014). As a unique mouse resource, together with the accumulated phenotypic information, the sequence of the 18 mouse genomes will serve as a basis for understanding trait differences. They can be also used for fine mapping known QTL or identifying new QTL on mouse Chr 1, and will help dissect the genetic basis of complex traits.

## Supplementary Material

Supplemental Material
