# Supplementary material for: Genome Sequencing of Chromosome 1 Substitution Lines Derived from Chinese Wild Mice Revealed a Unique Resource for Genetic Studies of Complex Traits
Source: G3 (Bethesda). 2016 Sep 6;6(11):3571–80. doi: 10.1534/g3.116.033902 (PMC5100856; doi:10.1534/g3.116.033902)
Supplement: Supplemental Material [file supp_6_11_3571__index.html]

Genome Sequencing of Chromosome 1 Substitution Lines Derived from Chinese Wild Mice Revealed a Unique Resource for Genetic Studies of Complex Traits — Supplemental Material 

# Genome Sequencing of Chromosome 1 Substitution Lines Derived from Chinese Wild Mice Revealed a Unique Resource for Genetic Studies of Complex Traits

## Supplemental Material for Xu, *et al*, 2016

**Files in this Data Supplement:**

- Figure S1 - Distribution of differentially expressed genes between B6 and HZ mice. (.tif, 9.95 KB)
- Table S1 - Summarization of genome sequencing, quality control, and read mapping. (.xlsx, 11 KB)
- Table S2 - Homozyosity rate of SNPs identified on Chr 1 for each line. (.xlsx, 11 KB)
- Table S3 - The amount of SNPs and indels discovered on Chr 1 and SNP density in 1kb from MGP sequenced 36 inbred strains. (.xlsx, 11 KB)
- Table S4 - List of frameshift variants across C1SLs. (.xlsx, 31 KB)
- Table S5 - List of stop lost or gain variants across C1SLs. (.xlsx, 23 KB)
- Table S6 - List of missense variants with deleterious effects across C1SLs. (.xlsx, 183 KB)
- Table S7 - Genes annotated to KEGG pathways. (.xlsx, 18 KB)
- Table S8 - Interspecies introgression regions in mouse Chr 1. (.xlsx, 11 KB)
- Table S9 - Percentage of analyzed 10kb blocks for inferring subspecies origin. (.xlsx, 12 KB)
- Table S10 - Regions on Chr 1 replaced by B6 sequences. (.xlsx, 10 KB)
