# Supplementary figures and images for: Genome Sequencing of Chromosome 1 Substitution Lines Derived from Chinese Wild Mice Revealed a Unique Resource for Genetic Studies of Complex Traits
Source: G3 (Bethesda). 2016 Sep 6;6(11):3571–80. doi: 10.1534/g3.116.033902 (PMC5100856; doi:10.1534/g3.116.033902)

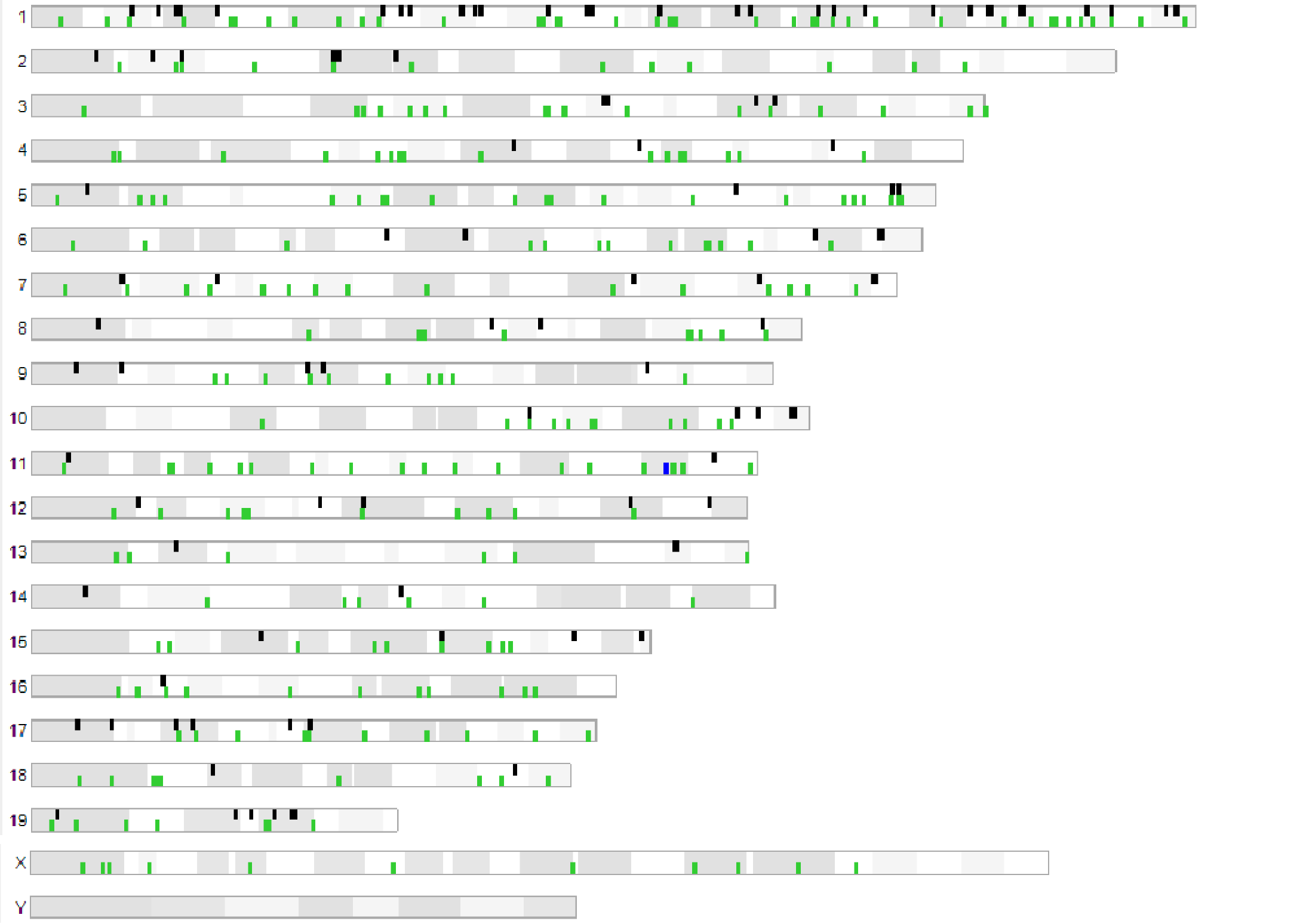

Supplement: Supplemental Material [file supp_g3.116.033902_FigureS1.tif]
