# Supplementary material for: Genome Sequencing of Chromosome 1 Substitution Lines Derived from Chinese Wild Mice Revealed a Unique Resource for Genetic Studies of Complex Traits
Source: G3 (Bethesda). 2016 Sep 6;6(11):3571–80. doi: 10.1534/g3.116.033902 (PMC5100856; doi:10.1534/g3.116.033902)
Supplement: Supplemental Material [file supp_g3.116.033902_TableS7.docx]

Table S7. Genes annotated to KEGG pathways. (.xlsx, 18 KB)

<http://www.g3journal.org/lookup/suppl/doi:10.1534/g3.116.033902/-/DC1/TableS7.xlsx>
